# Advances in Radiopharmaceutical Sciences for Vascular Inflammation Imaging: Focus on Clinical Applications

**DOI:** 10.3390/molecules26237111

**Published:** 2021-11-24

**Authors:** Kevin Prigent, Jonathan Vigne

**Affiliations:** 1 CHU de Caen Normandie, Department of Nuclear Medicine, Normandie Université, UNICAEN, 14000 Caen, France; prigent-k@chu-caen.fr; 2 CHU de Caen Normandie, Department of Pharmacy, Normandie Université, UNICAEN, 14000 Caen, France; 3 UNICAEN, INSERM U1237, Etablissement Français du Sang, Physiopathology and Imaging of Neurological Disorders (PhIND), Cyceron, Institut Blood and Brain @ Caen-Normandie (BB@C), Normandie University, 14000 Caen, France

**Keywords:** nuclear medicine, inflammation, atherosclerosis, large vessel vasculitis, [^18^F]fluorodeoxyglucose, [^18^F]fluoromethylcholine, [^68^Ga]Ga-DOTA-TATE

## Abstract

Biomedical imaging technologies offer identification of several anatomic and molecular features of disease pathogenesis. Molecular imaging techniques to assess cellular processes in vivo have been useful in advancing our understanding of several vascular inflammatory diseases. For the non-invasive molecular imaging of vascular inflammation, nuclear medicine constitutes one of the best imaging modalities, thanks to its high sensitivity for the detection of probes in tissues. 2-[^18^F]fluoro-2-deoxy-d-glucose ([^18^F]FDG) is currently the most widely used radiopharmaceutical for molecular imaging of vascular inflammatory diseases such as atherosclerosis and large-vessel vasculitis. The combination of [^18^F]FDG and positron emission tomography (PET) imaging has become a powerful tool to identify and monitor non-invasively inflammatory activities over time but suffers from several limitations including a lack of specificity and avid background in different localizations. The use of novel radiotracers may help to better understand the underlying pathophysiological processes and overcome some limitations of [^18^F]FDG PET for the imaging of vascular inflammation. This review examines how [^18^F]FDG PET has given us deeper insight into the role of inflammation in different vascular pathologies progression and discusses perspectives for alternative radiopharmaceuticals that could provide a more specific and simple identification of pathologies where vascular inflammation is implicated. Use of these novel PET tracers could lead to a better understanding of underlying disease mechanisms and help inform the identification and stratification of patients for newly emerging immune-modulatory therapies. Future research is needed to realize the true clinical translational value of PET imaging in vascular inflammatory diseases.

## 1. Introduction

Inflammatory disorders can affect virtually any organ and are a frequent cause of morbidity. The ability to image inflammatory processes in vivo can improve our understanding of the pathophysiology underlying various diseases including cancer, atherosclerosis, and neurodegenerative diseases. Intensive preclinical and translational research has been and has currently led to a deciphering of the involvement of the immune system in disease pathophysiology, quantify the course of a disease, and monitoring therapeutic interventions over time. Biomedical imaging enables the in vivo visualization of different morphologic features mainly using X-ray computed tomography (CT) and magnetic resonance imaging (MRI). However, these imaging modalities provide only structural information that is not sufficient to characterize inflammatory processes. Other imaging approaches called molecular imaging can take this information a step further, showing the activity of specific markers in vivo and how their location changes over time [1]. Advances in experimental and clinical molecular imaging largely contributed to a more comprehensive management of cancer and are likely to improve the understanding of non-oncological biological processes, including inflammatory diseases. For the in vivo detection, quantification, localization and monitoring of biological processes implicated in inflammatory disorders, nuclear imaging technologies appears as the most effective approach thanks to their high sensitivity to detect radiopharmaceutical probes in tissues [2]. Inflammation involves different potential targets related to its subprocesses such as: the surface expression of endothelial adhesion molecules [3,4,5], the platelet adhesion and the activation of leukocytes resulting in leukocyte transmigration into tissues [6,7,8]. These targets may represent a wide range of biomarkers that could be detected non-invasively using molecular imaging especially nuclear imaging that represent the most developed modality in the clinics able to image processes occurring at the molecular level [9].

It especially plays a major role in the outcomes of atherosclerosis that is both the most frequent cause of myocardial infarction (MI) and ischemic stroke (IS). Atherosclerosis is characterized by the accumulation of lipids, inflammatory cells and connective tissue within the arterial wall forming plaques that can become life-threatening when they rupture. It is now well-established that plaque formation is a complex process and that plaque rupture cannot be anticipated by focusing on morphological changes, but is mainly the consequence of plaque vulnerability that is closely linked to various biological processes [10]. Thus, imaging of relevant biomarkers of vulnerable plaques constitutes a great need in cardiovascular medicine.

Large vessel vasculitis (LVV) is also an important inflammation mediated vascular disease in which a progressive arterial injury and accelerated coronary atherosclerosis occurs [11]. Biomedical imaging is a key component of the diagnostic and disease-monitoring pathways for these cardiovascular inflammatory diseases. Echocardiography, CT, MRI, and nuclear perfusion imaging are first-line examinations corresponding to morphological and functional imaging. However, molecular imaging—especially positron emission tomography (PET)—can also play a major role in investigating molecular pathophysiological processes that occur earlier than functional and morphological damages [12].

At the interface between molecular biology and imaging, molecular imaging approaches may also have the potential to improve the identification of patients at high risk for cardiovascular events thanks to its ability to detect specific biological signatures. Over the last decade, PET combined with computed tomography (PET/CT) has emerged as a reliable molecular imaging modality for the characterization of vascular inflammation thanks to its high sensitivity for the detection of radiopharmaceuticals.

This review aims to provide a focused update on recent radiopharmaceuticals research related to nuclear imaging of vascular inflammation. An overview of ongoing research focused on clinical applications will be developed herein:

First, current and emerging nuclear probes for the in vivo molecular imaging of vascular inflammation.

Second, clinical findings of nuclear medicine to identify and stratify vascular inflammation.

## 2. Current and Emerging Nuclear Probes for the In Vivo Molecular Imaging of Vascular Inflammation

In recent decades, embedding anti-atherosclerotic medication into the medical regime of “vulnerable patients” has reshaped the course of the disease and the concept of vulnerable plaque-related thrombosis, shifting towards plaque erosion initiated acute coronary syndrome. The development of state-of-the-art imaging techniques, which beyond the morphological signs of atherosclerosis are also able to detect changes in molecular activity, is of utmost importance. The inherent properties of nuclear medicine, and especially PET that can non-invasively image specific molecular targets, could fulfill these criteria.

Nuclear medicine is among the most well-established molecular imaging techniques available in clinics so far. Among nuclear medicine modalities, PET is widely preferred for molecular targets detection because of higher spatial resolution, allowing less partial volume effect, which is particularly relevant in the field of vascular imaging due to the small size of the vessel wall. Also, this nuclear imaging modality benefits from validated methods of quantification. PET requires the use of radiotracers that are molecules bearing a positron emitting radionuclide, allowing them to be tracked in vivo after injection by the detection of by-product annihilation gamma rays. While PET is extremely sensitive, as it allows the detection of probes up to picomolar concentration, it suffers from poor spatial resolution. Hence, PET scanners are mainly hybrid systems used to co-register PET images with CT or MRI for accurate anatomical localization. The great advantage of PET as a molecular imaging technique lies in its ability to target specific pathologic features or processes of interest. PET is based on the detection of commonly used positron emitting radionuclides carbon-11, nitrogen-13, oxygen-15 in dedicated PET centers and most importantly fluorine-18 that is of widespread used in nuclear medicine departments. In the meantime, radiometals with positron emission properties have emerged in clinical applications such as copper-64 (^64^Cu), gallium-68 (^68^Ga) and rubidium-82 [13].

### 2.1. Small Molecules-Based PET Probes for the In Vivo Molecular Imaging of Vascular Inflammation

Vascular imaging with PET emerged with the concept of atherosclerosis as an inflammatory disease. This was highlighted by the ability of 2-[^18^F]fluoro-2-deoxy-D-glucose ([^18^F]FDG) PET to detect areas of high glucose metabolism in atherosclerotic plaques, a hallmark of macrophages accumulation within atherosclerosis, particularly in high-risk plaques [14]. These findings were confirmed and characterized in numerous studies [9,15]. Nowadays, the two most studied applications of PET for vascular imaging are atherosclerosis and LVV [16]. However, because of avid physiological uptake of [^18^F]FDG by cardiac myocytes, coronary arteries imaging is hampered, despite appropriate protocols, in up to 50% of patients with this tracer [17,18]. Also, [^18^F]FDG raises specificity issues for vascular PET because signal may also be attributed to cells that are not implicated in the inflammatory response.

**Figure 1:**
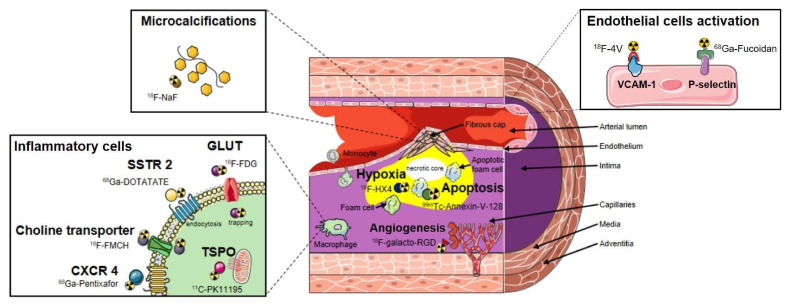
Vascular targets and corresponding examples of nuclear probes for the molecular imaging of atherosclerosis.

Consequently, alternative molecular targets and associated PET tracers for imaging vascular inflammation are being actively studied (Figure 1). Moreover, targets related to processes not directly linked to inflammation such as plaques microcalcifications, hypoxia or apoptosis are investigated. Vascular inflammation involves multiples cells of the immune system. Immune cells are associated with cytokine and receptors expression, resulting in a large spectra of potential imaging targets for new PET tracers [19]. For example, [^18^F]fluoromethylcholine ([^18^F]FMCH) that enters in phospholipids metabolism after passing through the choline transporter have shown potential to track macrophages accumulation in atherosclerotic plaques [20,21]. The somatostatin receptor SSTR type 2 is overexpressed by proinflammatory M1 macrophages and to a lesser extent by activated T-cells in atherosclerotic plaques. SSTR can be targeted using the octreotate derivative [^68^Ga]Ga-DOTA-TATE [22,23,24]. Also, the C-X-C chemokine receptor type 4 (CXCR4) corresponding to a chemokine receptor overexpressed by leukocytes including macrophages and lymphocytes [25] can be assessed by the [^68^Ga]Ga-pentixafor radioligand [26,27,28]. Other plasma membrane receptors largely expressed by macrophages have been targeted such as the C-type lectin CD206 commonly named mannose receptor. Radiopharmaceuticals presenting a large number of mannose moieties such as [^68^Ga]Ga-NOTA-neomannosylated human serum albumin ([^68^Ga]Ga-NOTA-MSA) [29] and [^99m^Tc]Tc-DTPA-mannosyl-dextran also known as [^99m^Tc]Tc-Tilmocept [30] have demonstrated their potential to detect macrophage-rich atherosclerotic plaques.

Activated macrophages have also been targeted through detection of the 18-kDa translocator protein (TSPO) expressed on the outer mitochondrial membrane using (R)-[^11^C]PK11195 ([^11^C]PK11195). In a study that analyzed culprit carotid plaques associated with stroke or transient ischemic attack in patients who underwent PET imaging with the TSPO radioligand [^11^C]PK11195, macrophage-rich plaque where identified by the radiotracer [30]. Such PET radioligand suffers from high interindividual variability in binding due to a genetic polymorphism in the TSPO gene (rs6971) and alternatives PET probes with low sensitivity to TSPO polymorphism are under development [31]. The turnover of macrophages is rapid in inflamed tissue and these cells have a relatively short life span, implicating intense DNA replication. Therefore, the non-invasive measure of plaque macrophages proliferation constitutes an interesting strategy to better characterize high-risk plaques. The PET thymidine analog named ^18^F-fluoro-3′-deoxy-3′-l-fluorothymidine ([^18^F]FLT) is a clinically approved probe indicated for the detection of proliferating cells in order to monitor treatment response in different oncological diseases [32,33]. The small molecule [^18^F]FLT is trapped intracellularly by phosphorylation allowing signal accumulation in areas of intense DNA replication. Its use has been investigated in the context of atherosclerosis and revealed that [^18^F]FLT avidly incorporates in plaques with a preponderant trapping from macrophages [34]. [^18^F]FLT is not specific for macrophages or hematopoietic progenitor cells, as parenchymal cells that proliferate in plaque may also contribute to the observed signal but may be an interesting tool to image proliferative activity in the vessel wall to evaluate treatment efficacy [35].

Biomarkers implicated in both plaque inflammation and related processes were also targeted for the development of PET probes. For instance, the integrin alpha-V-beta-3 (αvβ3) pathway is involved in intraplaque angiogenesis and inflammation and represents a promising target for molecular imaging in cardiovascular diseases such as atherosclerosis. αvβ3 integrin expression can be detected preclinically and clinically using peptides containing the RGD sequence derived PET probes such as the fluorinated and the gallium labeled [^18^F]galacto-RGD [36] and [^68^Ga]Ga-NOTA-RGD [37], respectively. Also, the clinical feasibility of imaging the fibroblast activation protein (FAP) in the human arterial vessel wall was recently demonstrated using the gallium-68-conjugated quinoline-based FAP inhibitor [^68^Ga]Ga-DOTA-FAPI-04 [38]. FAP is a membrane-bound, constitutively active serine protease expressed by activated fibroblasts in epithelial tumour stroma, arthritis, and wound healing, it presents the advantage to remain virtually undetectable in healthy tissues. FAP expression was demonstrated to be induced by macrophage-derived tumor necrosis factor alpha and to be associated with thin-cap fibroatheromata through its contribution to type I collagen breakdown in fibrous caps [39]. Furthermore, oxidized low-density lipoprotein that accumulates in vessel wall presents amyloid-like structural properties, and amyloid species have been recognized in human atherosclerotic plaques. Then, Hellberg et al. showed that [^18^F]Flutemetamol, a PET radiopharmaceutical known to accumulate in beta amyloid plaques (Aβ), presents a specific focal binding in human atherosclerotic plaque [40], the underlying mechanism is still unknown but the majority of Aβ deposition in human atherosclerotic plaques is suspected to be located in macrophages [41].

Interestingly, indications of the previously cited PET radiopharmaceuticals were initially dedicated to oncologic and neurologic explorations, and their repurposing potential in vascular imaging were subsequently assessed. The ability to use radiopharmaceuticals with already approved market authorization can facilitate the selection of interesting PET probes, as the presence of atherosclerotic plaques can be exhibited during a scan conducted for non-cardiovascular purposes. However, repurposing strategies may also question about target specificity given the heterogeneity of cell types implicated from oncological diseases to cardiovascular diseases. Currently, most of the PET probes used in clinical trials aiming at detecting vascular inflammation in the context of LVV or atherosclerosis (Table 1) were primarily dedicated to the field of oncology. However, preclinical studies interested in other targets to assess atherosclerotic lesions such as P-selectin [42], integrins and vascular cell adhesion molecule-1 [43,44] expressed on active endothelium and their corresponding radiolabeled high affinity ligands are under development and constitute a new vascular centered approach (Figure 1).

### 2.2. Nanoparticles-Based PET Probes for the In Vivo Molecular Imaging of Vascular Inflammation

Alternative nuclear in vivo imaging strategies based on the use of nanotechnologies to detect and quantify macrophages in ruptured and rupture-prone atherosclerotic plaques in humans are emerging. Nanoparticles have the great advantage to be easily tunable, and depending on their properties, they present longer biological half-lives than the small molecule–based PET radiopharmaceuticals discussed previously and then can be taken up by macrophages via phagocytosis. Indeed, macrophages are known to be among the first and primary cell types that process nanoparticles [68]. The main applications tested for translating macrophage-targeting nanoparticles to the clinics are mainly: cancer, atherosclerosis, myocardial infarction and stroke imaging, as macrophage infiltrates are common in these diseases [69]. Regarding atherosclerosis, different nanoplatforms were tested to image intraplaque macrophages but only a few have been evaluated in large animals and humans. One of the first tested in humans was composed of an iron oxide core solubilized with hydrophilic polymers, such as dextrans, with a mean diameter of 30 nm and called ultrasmall super paramagnetic particles of iron oxide (USPIOs) allowing to detect contrast by MRI [70]. However, the negative contrast on MRI induced by USPIOs and the other superparamagnetic agents is hard to interpret and displays relatively poor sensitivity and quantitative capabilities. To circumvent these limitations, the development of nanotracers transition to nanoparticles derivatized with radioisotopes, especially positron emitter with rather long half-lives such as copper-64 (t_1/2_ = 12.7 h) and zirconium-89 (^89^Zr) (t_1/2_ = 78.4 h). Then, based on the insights gained using USPIOs, 13 nm dextran nanoparticles (DNP) made from clinically approved components were developed and labeled with desferoxamine to allow radiolabeling with ^89^Zr to detect inflammatory leukocytes in murine atherosclerotic plaque using PET [71]. These radiolabeled nanoparticles were allowed to target inflammatory leukocytes, mostly macrophages, and allowed us to verify that ^89^Zr has a radioactive decay matching the in vivo kinetics of the 13-nm DNP. Therefore, the slow radioactive decay of ^89^Zr allows for delayed imaging that can be done after the nanoparticle has cleared from the blood pool, yielding a low-background signal.

In addition to their interesting pharmacokinetic and labeling properties, nanoparticles have the ability to be conjugated with various vectors, enabling more specific targeting. For instance, Woodard et al. tested a polymeric nanoparticle composed of high molecular weight polyethylene glycol graft copolymers containing a hydrophobic methyl methacrylate backbone, named comb. Comb nanoparticles were conjugated with the C-type atrial natriuretic factor (CANF) that is known to bind the natriuretic peptide clearance receptor that has been demonstrated to be a biomarker for atherosclerosis in both animal models and human coronary arteries as it is upregulated both in endothelium and in vascular smooth muscle cells [72]. Also, comb nanoparticles present reactive functional groups, allowing the attachment of diagnostic positron emitting moieties such as the 1,4,7,10-tetraazacyclododecane-1,4,7,10-tetraacetic acid (DOTA) chelate. [^64^Cu]Cu-DOTA-CANF-Comb were tested preclinically in apolipoprotein E–deficient (ApoE^−/−^) mice atherosclerosis model and showed a remarkable sensitivity to detect atherosclerotic plaques [66], enabling a transfer to clinical evaluation (Table 1). More recently, Comb nanoparticles were conjugated with the d-Ala-peptide T-amide (DAPTA-Comb) to target the chemokine receptor 5 (CCR5) implicated in the initiation and progression of atherosclerosis by mediating the trafficking of inflammatory cells. [^64^Cu]Cu-DOTA-DAPTA-Comb were tested both in an ApoE^–/–^ mouse model and in human carotid endarterectomy specimens. In the ApoE^–/–^ mouse model, results showed sensitive and specific detection of CCR5 in plaques not only along the progression of atherosclerotic lesions, but also during plaque regression and the human ex vivo experiment, which confirmed the potential of CCR5 targeting in human atherosclerosis [73].

Nahrendorf et al. reported the evaluation of a 20-nm spherical biocompatible nanoparticle composed of a macrophage sensor called Macrin to image and quantify macrophages content using [^64^Cu]-Macrin PET in various clinically relevant animal models of pulmonary and cardiovascular inflammation [67]. Data suggest that [^64^Cu]-Macrin PET is capable to assay tissue macrophages in various conditions including atherosclerosis and is currently tested in a clinical trial (Table 1). This report highlights that the composition of the nanoparticle itself is critical to achieve the desired targeting properties. Finally, the major advantages of nanoparticles derived imaging probes are to afford flexible design to control and modify the nanostructure size, morphology, composition, and surface properties through modular chemistry.

Different processes related to inflammation occurring in atherosclerotic plaques are represented (presence of inflammatory cells, angiogenesis, apoptosis, hypoxia, microcalcifications and endothelial cells activation). Numerous radiotracers able to track the presence of inflammatory cells in the vascular wall have been described. [^18^F]FDG is metabolically trapped after entering in the cell using glucose transporters (GLUT). Its uptake correlates with macrophages activity but suffers from a poor signal to noise ratio due to the concomitant glucose metabolism of non-inflammatory cells. It stimulates the need for more specific probes that are currently assessed such as: [^68^Ga]Ga-DOTA-TATE which accumulates in the cell by endocytosis after binding to the somatostatin receptor type 2 (SSTR 2); [^18^F]fluoromethylcholine ([^18^F]FMCH) that enters in phospholipids metabolism after passing through the choline transporter; [^11^C]PK11195 that target the 18-kDa translocator protein (TSPO) expressed on the outer mitochondrial membrane and 68Ga-pentixafor, which is a high affinity and selective ligand of the C-X-C chemokine receptor type 4 (CXCR4). Macrophages account for the majority of apoptotic cells within atherosclerotic plaques, and the programmed cell death of intraplaque macrophages, for instance, can be explored through use of the gamma emitter [^99m^Tc]Tc-Annexin-V-128. Inflammation is also a driver for a number of secondary hallmarks of plaque vulnerability. Intraplaque calcification (microcalcifications), which occurs in the fibrous cap, as it can be detected using [^18^F]fluoride (Na[^18^F]F), is initiated by inflammation and also thought to be part of a positive feedback mechanism. Also, angiogenesis contributes to plaque instability through intra-plaque haemorrhage and inflammatory cell infiltration, and is characterized by the overexpression of integrins (e.g., αvβ3 integrin) tracked by different peptides containing the RGD sequence (e.g., [^68^Ga]Ga-NOTA-RGD, [^18^F]galacto-RGD). The level of hypoxia is positively correlated with angiogenesis and increases in expanding plaques; it can be measured using a positron emitter such as the third-generation 2-nitroimidazole nucleoside analogue [^18^F]flortanidazole ([^18^F]HX4). Dysfunction of the endothelium may constitute an interesting biological pathway to track through the detection of adhesion molecules such as the vascular cell adhesion molecule-1 (VCAM-1) that can be targeted by a tetrameric linear peptide (TLP) radiolabeled with 4-[^18^F]fluorobenzaldehyde to form [^18^F]fluorobenzoyl-TLP ([^18^F]4V) or the P-selectin (targeted by [^68^Ga]Ga-fucoidan) expressed early at the surface of activated endothelial cells.

## 3. Clinical Findings of Nuclear Medicine to Identify and Stratify Vascular Inflammation

### 3.1. Atherosclerosis

#### 3.1.1. [^18^F]FDG PET/CT Imaging in Atherosclerosis

[^18^F]FDG PET/CT imaging is an established imaging in the inflammatory field [74] and has largely replaced the single photon emission computed tomography (SPECT) with the Gallium-67 radionuclide method previously used [75]. Thanks to a successful translational research, [^18^F]FDG is currently the most widely used probe in nuclear medicine corresponding to almost 90% of PET activity [76,77].

Preclinical studies demonstrated the interest of this tracer in the cardiovascular field [78]. First, in the 1990s, autoradiography showed a correlation in mice tumor between granulation tissue including macrophages from the tumor and [^18^F]FDG uptake [79]. Otherwise, GLUT receptors overexpression to allow [^18^F]FDG internalization was found in inflammatory preclinical model [80] similarly to the mechanism described as in cancer cells [81]. In the 2000s, a higher gamma counting after [^18^F]FDG injection assessed with positron-sensitive-fiberoptic probe was found in an inflammatory atherosclerosis rabbit model with a very strong correlation between histopathologic findings and radioactivity corresponding to smooth muscle cells and macrophages [82]. Both are the two main plaque components and macrophages is well known to be correlated with plaque rupture [83]. Macrophages release metalloprotease, resulting in fibrous cap degradation and release cytokine such as inter-leukin1 attracting other monocytes with a higher inflammatory state and higher cardiovascular risk [84]. To summarize, the more macrophages infiltrated, the thinner the fibrous cap and the higher the risk of plaque rupture. [^18^F]FDG uptake is linked to macrophage density and PET imaging confirmed these results in atherosclerosis preclinical model [85].

**Figure 2:**
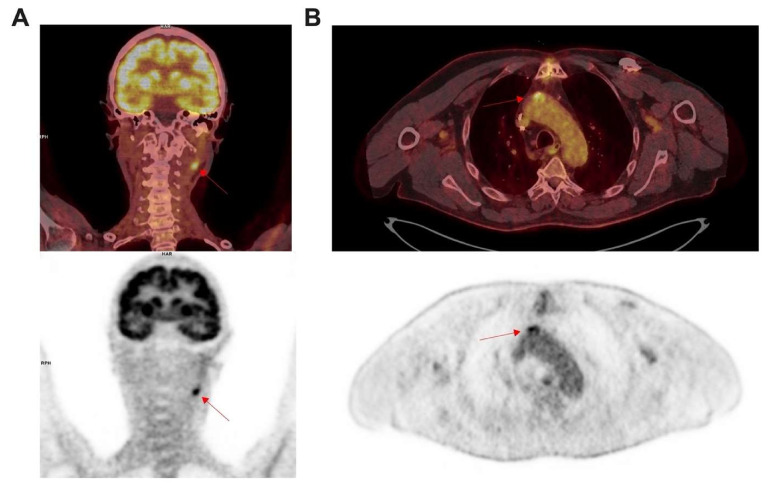
Incidental findings from atherosclerosis in [^18^F]FDG PET/CT imaging. Left panel (**A**) show coronal images performed for a lung cancer disease in 75 years old women. Intense focal uptake was found ((**A**) red arrow) corresponding to an inflammatory atherosclerosis process of left internal carotid. Right panel (**B**) show axial images performed to characterize lung nods in 76 years old patient also followed for a diabetes, tabagism and coronary bypass. Intense focal uptake was found in the ascending aorta ((**B**) red arrow) corresponding to an inflammatory process in an atherosclerosis plaque with mineralization.

The clinical translation of the previous findings was quickly performed, especially because it requires an already approved radiopharmaceutical for human use. A first clinical trial showed the feasibility of [^18^F]FDG PET/CT imaging to assess symptomatic atherosclerosis in carotid plaques [14]. A higher uptake was found in culprit lesion. Thereafter, many clinical studies confirmed the correlation between [^18^F]FDG uptake in patients and macrophage density from carotid endarterectomy specimens [86,87]. Inflammation is a hallmark of unstable atherosclerotic plaques leading to a higher uptake in culprit lesions [88]. Furthermore, [^18^F]FDG uptake was found to be well correlated to the high-risk plaques rupture in arteries assessed on CT [89], as well on MRI [90], and was able to predict cardio-vascular risk [91]. Figure 2 shows incidental findings of atherosclerosis plaques located in the carotid artery or in the aorta that a nuclear physician can fortuitously detect in [^18^F]FDG PET/CT from patients referred for oncological indications. Prospective studies showed [^18^F]FDG uptake as a predictor of the recurrence of transient ischemia or stroke in carotid stenosis [92,93]. This is a promising imaging method to improve management to select patient for endarterectomy surgery or therapy monitoring such as statin with a good reproducibility [94,95,96,97,98]. Statin therapy produced significant rapid dose-dependent reductions in [^18^F]FDG uptake that may represent changes in atherosclerotic plaque inflammation [99]. Recently, metabolic and enzyme analysis from symptomatic plaques revealed a strong association with inflammation and higher glycolysis [100] in line with all previous studies. Consequently, the CANTOS study confirmed the efficacy regarding the rate of cardiovascular events under anti-interleukin 1b (Canakinumab) in patients with previous MI [101]. Therefore, inflammation seems unavoidable in atherosclerosis and [^18^F]FDG appeared to be an efficient biomarker to study for diagnostic, monitoring and stratification for patient management. However, an important limitation of [^18^F]FDG is its relative non-specific uptake for inflammation in plaques. Consequently, there is a need for more specific tracers allowing for a more specific detection of inflammatory activities in the vessel wall than [^18^F]FDG.

PET/CT images performed for a lung cancer disease in 75 year old women. Intense focal uptake was found (A; red arrow) corresponding to an inflammatory atherosclerosis process of left internal carotid. Right panel (B) show axial [^18^F]FDG PET/CT images performed to characterize lung nods in a 76 year old patient also followed for a diabetes, tabagism and coronary bypass. Intense focal uptake was found in the ascending aorta (B; red arrow), corresponding to an inflammatory process in an atherosclerosis plaque with mineralization.

#### 3.1.2. [^18^F]FMCH PET/CT Imaging in Atherosclerosis

Lipid accumulation and oxidation is essential to atherosclerosis pathogenesis to trigger inflammation and macrophage recruitment in the plaques. Activated macrophages need choline for the synthesis of phosphatidylcholine [102], a major phospholipid acting as a structural building block of biological membranes and as a second messenger implicated in cell surface receptors regulation [103]. Choline is taken up by specific transporter then metabolized by choline kinase to be finally incorporated in a cell’s membrane [104,105]. A fluorinated choline derived PET radiopharmaceutical, named [^18^F]FMCH, was first introduced in prostate cancer and brain imaging [106,107]. [^18^F]FMCH uptake reflects an increased activity of choline kinase or an upregulation of transporter reflecting cell activity, and in particular macrophage activity in atherosclerosis. Nowadays, we know that the [^18^F]FMCH uptake by intraplaque proliferating macrophages [108] is correlated to vascular inflammation [20].

Wyss et al. demonstrated in a rat model of thigh muscle infection that abscess was avid of [^18^F]FMCH and [^18^F]FDG, corresponding to granulocyte and macrophage activities [108]. In atherosclerotic apolipoprotein E-deficient mice (ApoE^−/−^), Matter et al. confirmed that [^18^F]FMCH uptake was correlated to macrophage density, with a better sensitivity for [^18^F]FMCH compared to [^18^F]FDG and concluded that [^18^F]FMCH identifies murine plaques better than [^18^F]FDG using ex vivo imaging [20].

**Figure 3:**
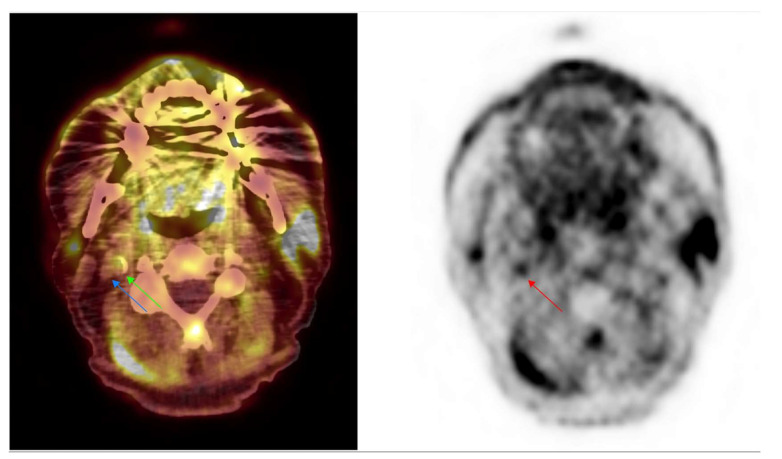
Incidental findings from atherosclerosis in [^18^F]FMCH PET/CT imaging. Axial [^18^F]FMCH PET/CT performed in a 66 year old patient to assess prostate adenocarcinoma disease. Images showed an intense focal uptake (red arrow) on PET imaging with an hyperdensity on PET/CT (green arrow) corresponding to an atherosclerosis plaque with mineralization from the right internal carotid. The internal vein is shown (blue arrow).

Numerous retrospective studies showed [^18^F]FMCH uptake in arteries and mainly in the mineralization process [21,61] (Figure 3). In a prospective study in 10 patients after acute cerebrovascular event who underwent [^18^F]FMCH PET/CT to assess maximum uptake in ipsilateral symptomatic carotid plaques and contralateral asymptomatic carotid arteries before carotid endarterectomy, Vöö et al. showed an increased uptake in culprit lesion with a correlation with macrophages amount as attested by immunohistological analysis [59]. [^18^F]FMCH displays interesting properties compared to [^18^F]FDG; on one hand, it does not require fasting and complex patient preparation with a low-carb/high-fat meal the day before PET; on the other hand, it does not present significant myocardial uptake, allowing for a better assessment of coronary arteries. Moreover, the quick clearance results in a better target to background ratio [109], with a shorter image acquisition time since the steady state is reached at 20 min after injection. However, [^18^F]FMCH is not yet approved in cardiovascular indications and high liver uptake may hamper assessment of the right coronary artery.

#### 3.1.3. [^68^Ga]Ga-DOTA-TATE PET/CT Imaging in Atherosclerosis

Numerous studies and reviews from preclinical to clinical applications have been published on the use of ^68^Ga labeled somatostatin analogues [22,23,110]. Compared to ^67^Ga, ^68^Ga offers many advantages such as the availability of an on-site generator allowing in-house labeling, allowing PET imaging, as it is a positron emitter and a shorter image acquisition duration. Among Gallium-68 labelled conjugate, there is an increasing use for the somatostatin analogues (SST) targeting G-protein-coupled receptor somatostatin receptor subtype-2 (SST2R) such as [^68^Ga]Ga-DOTA-TATE. [^68^Ga]Ga-DOTA-TATE is a selective radiopharmaceutical binding to somatostatin receptors subtype-2 (SSTR-2) that has been developed in the field of oncology, especially in neuroendocrine tumors with higher performance compared to other radiopharmaceuticals [111]. SST is a peptide hormone implicated in the regulation of the endocrine system isolated for the first time from ovine hypothalamus. It is particularly secreted by endocrine like cells and neurons [112]. SST2R is expressed by cells producing SST, including some inflammatory cells fostering its use to detect vascular inflammation, due to its specific uptake in macrophages or monocytes.

Preclinical studies based on autoradiography and PET imaging targeting SST2R showed an uptake in macrophage from unstable plaques in mice atherosclerosis model such as ApoE^−/−^ mice [113,114,115]. Nowadays, we know that SST2R is overexpressed by activated macrophages [116,117], playing an important role in early atherosclerosis. Since [^68^Ga]Ga-DOTA-TATE binds with high affinity to SSTR2 receptor overexpressed by macrophages and lymphocytes as well, inflammation findings are well reported as a source of pitfalls in the neuroendocrine tumor assessment [111]. Otherwise, [^68^Ga]Ga-DOTA-TATE demonstrated promising findings in cardiovascular inflammation. In many studies, atherosclerosis plaques findings were reported with a higher prevalence in patients with higher cardiovascular risk [22,118,119,120,121,122]. Some studies have specifically assessed its role in atherosclerotic plaques from carotid or coronary artery, resulting in a higher signal found in culprit lesion compared to stable lesion [23,123,124]. Lee et al. again demonstrated the correlation between vascular uptake and patient’s Framingham risk score [125]. Tarkin et al. confirmed in a prospective study in patients with acute coronary syndrome, transient IS, that high target SST2R was directly linked to proinflammatory M1 macrophages in atherosclerotic plaques using PET imaging and autoradiography. They also showed that vascular uptake was predictive of cardio-vascular risk with a higher accuracy for [^68^Ga]Ga-DOTA-TOC than for [^18^F]FDG. Moreover, a good correlation was found in vascular wall between the uptake of [^18^F]FDG and [^68^Ga]Ga-DOTA-TATE. Advantages of [^68^Ga]Ga-DOTA-TATE over [^18^F]FDG is the better signal to background ratio in vascular wall and the absence of spill-over from myocardium uptake especially to assess coronary arteries [23]. DOTA-TATE can also be radiolabeled with Copper-64 characterized by a longer half-life and shorter positron range but lower positron abundance and longer scanning duration compared to ^68^Ga [126]. Further studies are necessary to better understand the role of SST derived PET probes in vascular inflammation, and nuclear medicine physician should be aware of these cardiovascular findings using [^68^Ga]Ga-DOTA-TATE.

### 3.2. Large Vessel Vasculitis

LVV includes two main entities: Takayasu disease and Giant cell arteritis (GCA) [127,128]. Early symptoms of Takayasu disease are non-specific and include constitutional and musculoskeletal symptoms. At a later stage, vascular complications involving the aorta and its main branches become manifest. GCA mainly involves large- and medium-sized arteries, particularly the branches of the proximal aorta, and including the temporal arteries. GCA may involve smallest vessels as extra cranial branches also. Inflammatory processes occurring at the vascular level is an important issue addressed in the management of these patients.

#### [^18^F]FDG PET/CT Imaging in Large Vessel Vasculitis

**Figure 4:**
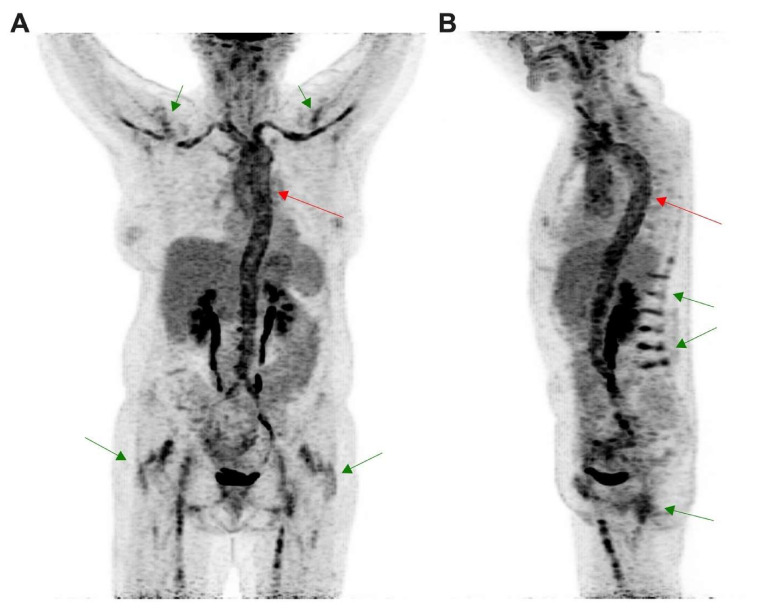
[^18^F]FDG PET/CT was performed in a 64 year old patient for a suspicion of giant cell arteritis. (**A**) Coronal and (**B**) sagittal images obtained from maximal intensity projection revealed intense uptake in the vascular wall from the whole aorta (red arrows) extended to axillary and femoral vessels, above the liver uptake, corresponding to an LVV. LVV was associated with patterns of polymyalgia rheumatica (green arrows) corresponding to interspinous bursitis ((**B**) green arrow) and also inflammation in hips and shoulders joints ((**A**) green arrow).

LVV can also be assessed by [^18^F]FDG imaging [129] with very good diagnostic performance [130,131,132,133]. Transmural inflammation precedes complications such as obstruction or aneurysm and dissection. In that way, [^18^F]FDG imaging allows us to detect vascular inflammation even in the absence of morphological evidence [134,135,136,137]. As shown in Figure 4, this is probably the most sensitive method to determine the inflammatory activity of the disease [138]. Although there is a need to harmonize the interpretation criteria, currently, visual analysis is advised using grading scale (vascular to liver uptake) [129]. Moreover, its whole-body scan nature may even detect inflammation of a small vessel using digital PET and recent improvements in spatial resolution to reduce partial volume effects [139]. Beyond its diagnostic role, [^18^F]FDG PET/CT may also help to monitor treatment efficacy in LVV patients [140].

Several limitations and precautions are important to address before performing [^18^F]FDG PET/CT imaging of LVV. First, before the injection of [^18^F]FDG, patients need to fast at least 6 h to prevent tracer uptake in peripheral muscles. It can be relevant to assess coronary lesions in atherosclerotic as well as potential iatrogenic toxicity met in vasculitis treatment [141]. To assess myocardial involvement and coronary arteries, patient must ideally fast at least 12 h to suppress physiological myocardial uptake. To ensure a metabolic switch from sugar to lipid in cardiomyocyte, an unfractionated heparin dose can be administered before [^18^F]FDG injection in addition to a fat diet with low carbohydrate the day before [142,143]. While an important proportion of patients susceptible to have vascular inflammation are likely to be diabetics, effort must be made to control glycemia around 126 mg/dL to avoid degradation of signal-to-noise ratio [129]. Other limits are linked to a high uptake of [^18^F]FDG in healthy organs such as brain and Waldeyer ring that can hamper detection of small inflammatory vessels in these areas [144]. In vasculitis context, glucocorticoids are frequently prescribed which may impact the performance of [^18^F]FDG PET/CT imaging. Glucocorticoids are responsible for significantly lower the standardized uptake value (SUV) after 10 days of treatment [51,52,145]. However, it should be emphasized that currently the diagnosis is mainly based on visual analysis in LVV patients, and the use of SUV is not recommended. Furthermore, grading scales could be impacted since glucocorticoids may increase liver uptake resulting in false negative interpretations [137]. Finally, experimentations in atherosclerosis plaques obtained from endarterectomies or autopsies showed that [^18^F]FDG uptake resulted from various cells types [146]. Indeed, experimentations showed that [^18^F]FDG uptake in activated macrophages resulted mostly from hypoxic conditions than inflammatory stimulation contrary to vascular smooth muscle cells and activated endothelial cells. The development of more specific radiotracers dedicated to LVV evaluation is necessary and the need is growing. The ideal radiotracer would be able to discriminate inflammation from infection and to differentiate acute from chronic vascular inflammation. For instance, to be more specific, macrophages targeting could be relevant since they are highly abundant in vascular inflammation. Among its receptors SSTR2 is overexpressed [23,115], but to the best of our knowledge, evidences in LVV are, for the moment, scarce.

## 4. Conclusions

Current approaches to personalized medicine have resulted in advanced imaging tools for the evaluation of vascular inflammation, especially in the context of atherosclerosis. Nuclear imaging techniques will further enhance our understanding of vascular inflammation related disease mechanisms. PET imaging allows for the direct visualization of metabolic processes, including plaque inflammation, bone formation and macrophage activity, which is already widely studied in humans. Intense development of PET probes is currently ongoing and will hopefully implement a toolbox of nuclear probes able to explore new molecular targets.

## Figures and Tables

**Table 1:** Registered clinical trials evaluating nuclear probes to image vascular inflammation in large vessel vasculitis and atherosclerosis. [^18^F]FDG: 2-[^18^F]fluoro-2-deoxy-d-glucose; SSTR2: somatosatin receptor 2; ^68^Ga: Gallium-68; ^64^Cu: Copper-64; [^18^F]FMCH: [^18^F]fluoromethylcholine; [^68^Ga]Ga-NOTA-MSA: [^68^Ga]Ga-NOTA-neomannosylated human serum albumin; anti-MMR-VHH2: nanobody targeting macrophage mannose receptor; [^11^C]PBR28: [^11^C]*N*-acetyl-*N*-(2-methoxybenzyl)-2-phenoxy-5-pyridinamine); [^18^F]RGD-K5: ^18^F-fluorination of an arginine-glycine-aspartic acid derivated peptide targeting integrin αvβ3; FAP: fibroblast activating protein; FAPI: fibroblast activating protein inhibitor; CCR2: C–C chemokine receptor 2; ECL1i: extracellular loop 1 inverso; Aβ: beta amyloid deposits.

Molecular Target	Nuclear Probes	Information	National Clinical Trial Number	References
**Large vessel vasculitis**				
GLUT transporters	[^18^F]FDG	Macrophage metabolism	NCT03914248 NCT04204876 NCT03765424 NCT03550781 NCT04888221 NCT01588483 NCT00744952 NCT03285945	[11,45,46,47,48,49,50,51,52]
SSTR2	[^68^Ga]Ga-DOTA-TATE [^18^F]fluoroethyltriazole-octreotate	Macrophage activity	NCT04071691	[53]
	[^68^Ga]Ga-DOTA-TATE		NCT03812302	
TSPO	[^11^C]PK11195	Macrophage activity	NCT01878721	[54]
**Atherosclerosis**				
GLUT transporters	[^18^F]FDG	Macrophage metabolism	NCT04181996 NCT00633022 NCT01341730 NCT01186666 NCT02162303 NCT03215550 NCT04505865 NCT04350216	[55,56,57,58]
Choline transporter	[^18^F]FMCH	Macrophage activity	NCT03252990 NCT02640313	[59,60,61]
SSTR2	[^68^Ga]Ga-DOTA-TATE	Macrophage activity	NCT04043377 NCT04073810 NCT02021188	[22,23,62]
Mannose receptors	[^68^Ga]Ga-NOTA-MSA [^99m^Tc]Tc-Tilmanocept [^68^Ga]Ga-NOTA-anti-MMR-VHH2	Macrophage activity	NCT01893489 NCT01889693 NCT02542371 NCT04758650	[29,63]
TSPO	[^11^C]PBR28 [^11^C]PK11195	Macrophage activity	NCT00547976	[64]
Integrins	[^18^F]RGD-K5 [^68^Ga]Ga-NOTA-RGD	Neoangiogenesis and macrophage activity	NCT03364270	[37]
FAP	[^68^Ga]Ga-DOTA-FAPI-04	Proinflammatory macrophages and type I collagen breakdown in fibrous caps	NCT05036759	[39]
CCR2	[^64^Cu]Cu-DOTA-ECL1i	Pro-inflammatory macrophages	NCT04537403	[65]
Aβ	[^18^F]flutemetamol	Aβ deposition in human atherosclerotic plaques	NCT03291093	[40]
NPR-C	[^64^Cu]Cu-DOTA-CANF-Comb	Endothelial and vascular smooth muscle cells activation	NCT02498379 NCT02417688	[66]
-	[^64^Cu]Cu-macrin	Macrophage phagocytic activity	NCT04843891	[67]

## Data Availability

Not applicable.

## References

[1] Weissleder R., Pittet M.J. (2008). Imaging in the era of molecular oncology. Nature.

[2] Hammoud D.A. (2016). Molecular Imaging of Inflammation: Current Status. J. Nucl. Med. Soc. Nucl. Med..

[3] Vigne J., Bay S., Aid-Launais R., Pariscoat G., Rucher G., Sénémaud J., Truffier A., Anizan N., Even G., Ganneau C. (2019). Cleaved CD31 as a target for in vivo molecular imaging of inflammation. Sci. Rep..

[4] Vigne J., Cognet T., Guedj K., Morvan M., Merceron O., Louedec L., Choqueux C., Nicoletti A., Escoubet B., Chaubet F. (2019). Early Detection of Localized Immunity in Experimental Autoimmune Myocarditis Using [^99m^Tc]Fucoidan SPECT. Mol. Imaging Biol..

[5] Troncoso M.F., Ortiz-Quintero J., Garrido-Moreno V., Sanhueza-Olivares F., Guerrero-Moncayo A., Chiong M., Castro P.F., García L., Gabrielli L., Corbalán R. (2021). VCAM-1 as a predictor biomarker in cardiovascular disease. Biochim. Biophys. Acta Mol. Basis Dis..

[6] Herter J., Zarbock A. (2013). Integrin Regulation during Leukocyte Recruitment. J. Immunol..

[7] Daniel A.E., van Buul J.D. (2013). Endothelial junction regulation: A prerequisite for leukocytes crossing the vessel wall. J. Innate Immun..

[8] Esmon C.T. (2005). The interactions between inflammation and coagulation. Br. J. Haematol..

[9] Libby P., Loscalzo J., Ridker P.M., Farkouh M.E., Hsue P.Y., Fuster V., Hasan A.A., Amar S. (2018). Inflammation, Immunity, and Infection in Atherothrombosis: JACC Review Topic of the Week. J. Am. Coll. Cardiol..

[10] Choudhury R.P., Fuster V., Fayad Z.A. (2004). Molecular, cellular and functional imaging of atherothrombosis. Nat. Rev. Drug Discov..

[11] Weyand C.M., Goronzy J.J. (2003). Medium- and large-vessel vasculitis. N. Engl. J. Med..

[12] Zhou W., Dey A., Manyak G., Teklu M., Patel N., Teague H., Mehta N.N. (2020). The application of molecular imaging to advance translational research in chronic inflammation. J. Nucl. Cardiol..

[13] Coenen H.H., Ermert J. (2021). Expanding PET-applications in life sciences with positron-emitters beyond fluorine-18. Nucl. Med. Biol..

[14] Rudd J.H.F., Warburton E.A., Fryer T.D., Jones H.A., Clark J.C., Antoun N., Johnström P., Davenport A.P., Kirkpatrick P.J., Arch B N. (2002). Imaging atherosclerotic plaque inflammation with [^18^F]-fluorodeoxyglucose positron emission tomography. Circulation.

[15] Joseph P., Tawakol A. (2016). Imaging atherosclerosis with positron emission tomography. Eur. Heart J..

[16] Ćorović A., Wall C., Mason J.C., Rudd J.H.F., Tarkin J.M. (2020). Novel Positron Emission Tomography Tracers for Imaging Vascular Inflammation. Curr. Cardiol. Rep..

[17] Cheng V.Y., Slomka P.J., Meunier L.L., Tamarappoo B.K., Nakazato R., Dey D., Berman D.S. (2012). Coronary Arterial ^18^F-FDG Uptake by Fusion of PET and Coronary CT Angiography at Sites of Percutaneous Stenting for Acute Myocardial Infarction and Stable Coronary Artery Disease. J. Nucl. Med. Soc. Nucl. Med..

[18] Joshi N.V., Vesey A.T., Williams M.C., Shah A.S.V., Calvert P.A., Craighead F.H.M., Yeoh S.E., Wallace W., Salter D., Fletcher A.M. (2014). ^18^F-fluoride positron emission tomography for identification of ruptured and high-risk coronary atherosclerotic plaques: A prospective clinical trial. Lancet.

[19] Vorster M., Maes A., van de Wiele C., Sathekge M. (2016). Gallium-68 PET: A Powerful Generator-based Alternative to Infection and Inflammation Imaging. Semin. Nucl. Med..

[20] Matter C.M., Wyss M.T., Meier P., Späth N., von Lukowicz T., Lohmann C., Weber B., de Molina A.R., Lacal J.C., Ametamey S.M. (2006). ^18^F-choline images murine atherosclerotic plaques ex vivo. Arterioscler. Thromb. Vasc. Biol..

[21] Bucerius J., Schmaljohann J., Böhm I., Palmedo H., Guhlke S., Tiemann K., Schild H.H., Biersack H.-J., Manka C. (2008). Feasibility of ^18^F-fluoromethylcholine PET/CT for imaging of vessel wall alterations in humans—First results. Eur. J. Nucl. Med. Mol. Imaging.

[22] Rominger A., Saam T., Vogl E., Übleis C., la Fougère C., Förster S., Haug A., Cumming P., Reiser M.F., Nikolaou K.L. (2010). In Vivo Imaging of Macrophage Activity in the Coronary Arteries Using ^68^Ga-DOTATATE PET/CT: Correlation with Coronary Calcium Burden and Risk Factors. J. Nucl. Med. Soc. Nucl. Med..

[23] Tarkin J.M., Joshi F.R., Evans N.R., Chowdhury M.M., Figg N.L., Shah A.V., Starks L.K., Martin-Garrido A., Manavaki R., Yu E. (2017). Detection of Atherosclerotic Inflammation by ^68^Ga-DOTATATE PET Compared to [^18^F]FDG PET Imaging. J. Am. Coll. Cardiol..

[24] Ambrosini V., Zompatori M., Luca F.D., Antonia D., Allegri V., Nanni C., Malvi D., Tonveronachi E., Fasano L., Fabbri M. (2010). ^68^Ga-DOTANOC PET/CT Allows Somatostatin Receptor Imaging in Idiopathic Pulmonary Fibrosis: Preliminary Results. J. Nucl. Med. Soc. Nucl. Med..

[25] Li X., Yu W., Wollenweber T., Lu X., Wei Y., Beitzke D., Wadsak W., Kropf S., Wester H.J., Haug A.R. (2019). [^68^Ga]Pentixafor PET/MR imaging of chemokine receptor 4 expression in the human carotid artery. Eur. J. Nucl. Med. Mol. Imaging.

[26] Schmid J.S., Schirbel A., Buck A.K., Kropf S., Wester H.-J., Lapa C. (2016). [^68^Ga]Pentixafor-Positron Emission Tomography/Computed Tomography Detects Chemokine Receptor CXCR4 Expression After Ischemic Stroke. Circ. Cardiovasc. Imaging.

[27] Thackeray J.T., Derlin T., Haghikia A., Napp L.C., Wang Y., Ross T.L., Schäfer A., Tillmanns J., Wester H.J., Wollert K.C. (2015). Molecular Imaging of the Chemokine Receptor CXCR4 After Acute Myocardial Infarction. JACC Cardiovasc. Imaging.

[28] Werner L., Guzner-Gur H., Dotan I. (2013). Involvement of CXCR4/CXCR7/CXCL12 Interactions in Inflammatory bowel disease. Theranostics.

[29] Kim E.J., Kim S., Seo H.S., Lee Y.J., Eo J.S., Jeong J.M., Lee B., Kim J.Y., Park Y.M., Jeong M. (2016). Novel PET Imaging of Atherosclerosis with ^68^Ga-Labeled NOTA-Neomannosylated Human Serum Albumin. J. Nucl. Med..

[30] Zanni M.V., Toribio M., Wilks M.Q., Lu M.T., Burdo T.H., Walker J., Autissier P., Foldyna B., Stone L., Martin A. (2017). Application of a Novel CD206^+^ Macrophage-Specific Arterial Imaging Strategy in HIV-Infected Individuals. J. Infect. Dis..

[31] Ramakrishnan N.K., Hird M., Thompson S., Williamson D.J., Qiao L., Owen D.R., Brooks A.F., Scott P.J.H., Bacallado S., O’Brien J.T. (2021). Preclinical evaluation of (*S*)-[^18^F]GE387, a novel 18-kDa translocator protein (TSPO) PET radioligand with low binding sensitivity to human polymorphism rs6971. Eur. J. Nucl. Med. Mol. Imaging.

[32] Chalkidou A., Landau D.B., Odell E.W., Cornelius V.R., O’Doherty M.J., Marsden P.K. (2012). Correlation between Ki-67 immunohistochemistry and ^18^F-fluorothymidine uptake in patients with cancer: A systematic review and meta-analysis. Eur. J. Cancer..

[33] Bading J.R., Shields A.F. (2008). Imaging of Cell Proliferation: Status and Prospects. J. Nucl. Med. Soc. Nucl. Med..

[34] Ye Y.-X., Calcagno C., Binderup T., Courties G., Keliher E.J., Wojtkiewicz G.R., Iwamoto Y., Tang J., Pérez-Medina C., Mani V. (2015). Imaging Macrophage and Hematopoietic Progenitor Proliferation in Atherosclerosis. Circ. Res..

[35] Binderup T., Duivenvoorden R., Fay F., van Leent M.M.T., Malkus J., Baxter S., Ishino S., Zhao Y., Sanchez-Gaytan B., Teunissen A.J.P. (2019). Imaging-assisted nanoimmunotherapy for atherosclerosis in multiple species. Sci. Transl. Med..

[36] Laitinen I., Saraste A., Weidl E., Poethko T., Weber A.W., Nekolla S.G., Leppänen P., Ylä-Herttuala S., Hölzlwimmer G., Walch A. (2009). Evaluation of alphavbeta3 integrin-targeted positron emission tomography tracer ^18^F-galacto-RGD for imaging of vascular inflammation in atherosclerotic mice. Circ. Cardiovasc. Imaging.

[37] Dietz M., Kamani C.H., Deshayes E., Dunet V., Mitsakis P., Coukos G., Lalonde M.N., Schaefer N., Prior J.O. (2021). Imaging angiogenesis in atherosclerosis in large arteries with ^68^Ga-NODAGA-RGD PET/CT: Relationship with clinical atherosclerotic cardiovascular disease. EJNMMI Res..

[38] Wu M., Ning J., Li J., Lai Z., Shi X., Xing H., Hacker M., Liu B., Huo L., Li X. (2021). Feasibility of in vivo Imaging of Fibroblast Activation Protein in Human Arterial Walls. J. Nucl. Med. Soc. Nucl. Med..

[39] Brokopp C.E., Schoenauer R., Richards P., Bauer S., Lohmann C., Emmert M.Y., Weber B., Winnik S., Aikawa E., Graves K. (2011). Fibroblast activation protein is induced by inflammation and degrades type I collagen in thin-cap fibroatheromata. Eur. Heart J..

[40] Hellberg S., Silvola J.M.U., Liljenbäck H., Kiugel M., Eskola O., Hakovirta H., Hörkkö S., Morisson-Iveson V., Hirani E., Saukko P. (2019). Amyloid-Targeting PET Tracer [^18^F]Flutemetamol Accumulates in Atherosclerotic Plaques. Molecules.

[41] Bucerius J., Barthel H., Tiepolt S., Werner P., Sluimer J.C., Wildberger J.E., Patt M., Hesse S., Gertz H.-J., Biessen E.A.L. (2017). Feasibility of in vivo ^18^F-florbetaben PET/MR imaging of human carotid amyloid-β. Eur. J. Nucl. Med. Mol. Imaging.

[42] Li X., Bauer W., Israel I., Kreissl M.C., Weirather J., Richter D., Bauer E., Herold V., Jakob P., Buck A. (2014). Targeting *P*-selectin by gallium-68-labeled fucoidan positron emission tomography for noninvasive characterization of vulnerable plaques: Correlation with in vivo 17.6T MRI. Arterioscler. Thromb. Vasc. Biol..

[43] Silvola J.M.U., Virtanen H., Siitonen R., Hellberg S., Liljenbäck H., Metsälä O., Ståhle M., Saanijoki T., Käkelä M., Hakovirta H. (2016). Leukocyte trafficking-associated vascular adhesion protein 1 is expressed and functionally active in atherosclerotic plaques. Sci. Rep..

[44] Haukkala J., Laitinen I., Luoto P., Iveson P., Wilson I., Karlsen H., Cuthbertson A., Laine J., Leppänen P., Ylä-Herttula S. (2009). ^68^Ga-DOTA-RGD peptide: Biodistribution and binding into atherosclerotic plaques in mice. Eur. J. Nucl. Med. Mol. Imaging.

[45] Imfeld S., Rottenburger C., Schegk E., Aschwanden M., Juengling F., Staub D., Recher M., Kyburz D., Berger C.T., Daikeler T. (2018). [^18^F]FDG positron emission tomography in patients presenting with suspicion of giant cell arteritis-lessons from a vasculitis clinic. Eur. Heart J. Cardiovasc. Imaging.

[46] Grayson P.C., Alehashemi S., Bs A.A.B., Civelek A.C., Cupps T.R., Kaplan M.J., Malayeri A.A., Merkel P.A., Rn E.N., Bluemke D.A. (2018). ^18^F-Fluorodeoxyglucose-Positron Emission Tomography as an Imaging Biomarker in a Prospective, Longitudinal Cohort of Patients with Large Vessel Vasculitis. Arthritis Rheumatol..

[47] Blockmans D., Coudyzer W., Vanderschueren S., Stroobants S., Loeckx D., Heye S., Ceuninck L.D., Marchal G., Bobbaers H. (2008). Relationship between fluorodeoxyglucose uptake in the large vessels and late aortic diameter in giant cell arteritis. Rheumatology.

[48] Stone J.H., Hoffman G.S., Merkel P.A., Min Y.I., Uhlfelder M.L., Hellmann D.B., Specks U., Allen N.B., Davis J.C., Spiera R.F. (2001). A disease-specific activity index for Wegener’s granulomatosis: Modification of the Birmingham Vasculitis Activity Score. International Network for the Study of the Systemic Vasculitides (INSSYS). Arthritis Rheum..

[49] Tso E., Flamm S.D., White R.D., Schvartzman P.R., Mascha E., Hoffman G.S. (2002). Takayasu arteritis: Utility and limitations of magnetic resonance imaging in diagnosis and treatment. Arthritis Rheum..

[50] Hoffman G.S., Merkel P.A., Brasington R.D., Lenschow D.J., Liang P. (2004). Anti-tumor necrosis factor therapy in patients with difficult to treat Takayasu arteritis. Arthritis Rheum..

[51] Fuchs M., Briel M., Daikeler T., Walker U.A., Rasch H., Berg S., Ng Q.K.T., Raatz H., Jayne D., Kötter I. (2012). The impact of ^18^F-FDG PET on the management of patients with suspected large vessel vasculitis. Eur. J. Nucl. Med. Mol. Imaging.

[52] Prieto-González S., García-Martínez A., Tavera-Bahillo I., Hernández-Rodríguez J., Gutiérrez-Chacoff J., Alba M.A., Murgia G., Espígol-Frigolé G., Sánchez M., Arguis P. (2015). Effect of glucocorticoid treatment on computed tomography angiography detected large-vessel inflammation in giant-cell arteritis. A prospective, longitudinal study. Medicine.

[53] Tarkin J.M., Wall C., Gopalan D., Aloj L., Manavaki R., Fryer T.D., Aboagye E.O., Bennett M.R., Peters J.E., Rudd J.H.F. (2020). Novel Approach to Imaging Active Takayasu Arteritis Using Somatostatin Receptor Positron Emission Tomography/Magnetic Resonance Imaging. Circ. Cardiovasc. Imaging.

[54] Pugliese F., Gaemperli O., Kinderlerer A.R., Lamare F., Shalhoub J., Davies A.H., Rimoldi O.E., Mason J., Camici P.G. (2010). Imaging of vascular inflammation with [^11^C]-PK11195 and positron emission tomography/computed tomography angiography. J. Am. Coll. Cardiol..

[55] Tardif J.-C., Kouz S., Waters D.D., Bertrand O.F., Diaz R., Maggioni A.P., Pinto F.J., Ibrahim R., Gamra H., Kiwan G.S. (2019). Efficacy and Safety of Low-Dose Colchicine after Myocardial Infarction. N. Engl. J. Med..

[56] Nidorf S.M., Eikelboom J.W., Budgeon C.A., Thompson P.L. (2013). Low-dose colchicine for secondary prevention of cardiovascular disease. J. Am. Coll. Cardiol..

[57] Elkhawad M., Rudd J.H., Sarov-Blat L., Cai G., Wells R., Davies L.C., Collier D.J., Marber M.S., Choudhury R.P., Fayad Z.A. (2012). Effects of p38 mitogen-activated protein kinase inhibition on vascular and systemic inflammation in patients with atherosclerosis. JACC Cardiovasc. Imaging.

[58] Choo E.H., Han E.J., Kim C.J., Kim S.H., Joo-Hyun O., Chang K., Seung K.B. (2018). Effect of Pioglitazone in Combination with Moderate Dose Statin on Atherosclerotic Inflammation: Randomized Controlled Clinical Trial Using Serial FDG-PET/CT. Korean Circ. J..

[59] Vöö S., Kwee R.M., Sluimer J.C., Schreuder F.H.B.M., Wierts R., Bauwens M., Heeneman S., Cleutjens J.P.M., van Oostenbrugge R.J., Daemen J.-W.H. (2016). Imaging Intraplaque Inflammation in Carotid Atherosclerosis with ^18^F-Fluorocholine Positron Emission Tomography-Computed Tomography: Prospective Study on Vulnerable Atheroma with Immunohistochemical Validation. Circ. Cardiovasc. Imaging.

[60] Kato K., Schober O., Ikeda M., Schäfers M., Ishigaki T., Kies P., Naganawa S., Stegger L. (2009). Evaluation and comparison of ^11^C-choline uptake and calcification in aortic and common carotid arterial walls with combined PET/CT. Eur. J. Nucl. Med. Mol. Imaging.

[61] Förster S., Rominger A., Saam T., Wolpers S., Nikolaou K., Cumming P., Reiser M.F., Bartenstein P., Hacker M. (2010). ^18^F-fluoroethylcholine uptake in arterial vessel walls and cardiovascular risk factors: Correlation in a PET-CT study. Nuklearmedizin.

[62] Tarkin J.M., Calcagno C., Dweck M.R., Evans N.R., Chowdhury M.M., Gopalan D., Newby D.E., Fayad Z.A., Bennett M.R., Rudd J.H.F. (2019). ^68^Ga-DOTATATE PET Identifies Residual Myocardial Inflammation and Bone Marrow Activation after Myocardial Infarction. J. Am. Coll. Cardiol..

[63] Senders M., Hernot S., Carlucci G., van de Voort J.C., Fay F., Calcagno C., Tang J., Alaarg A., Zhao Y., Ishino S. (2019). Nanobody-Facilitated Multiparametric PET/MRI Phenotyping of Atherosclerosis. JACC Cardiovasc. Imaging.

[64] Gaemperli O., Shalhoub J., Owen D., Lamare F., Johansson S., Fouladi N., Davies A.H., Rimoldi O.E., Camici P.G. (2012). Imaging intraplaque inflammation in carotid atherosclerosis with ^11^C-PK11195 positron emission tomography/computed tomography. Eur. Heart J..

[65] Luehmann H., Detering L., Gropler R.J., Liu Y. (2017). Abstract 20674: C–C Chemokine Receptor Type 2 (CCR2) Targeted PET Imaging of Early Atherosclerosis. Circ. Am. Heart Assoc..

[66] Woodard P.K., Liu Y., Pressly E.D., Luehmann H.P., Detering L., Sultan D.E., Laforest R., McGrath A.J., Gropler R.J., Hawker C.J. (2016). Design and Modular Construction of a Polymeric Nanoparticle for Targeted Atherosclerosis Positron Emission Tomography Imaging: A Story of 25% (64)Cu-CANF-Comb. Pharm. Res..

[67] Nahrendorf M., Hoyer F.F., Meerwaldt A.E., van Leent M.M., Senders M.L., Calcagno C., Robson P.M., Soultanidis G., Pérez-Medina C., Teunissen A.J. (2020). Imaging Cardiovascular and Lung Macrophages with the Positron Emission Tomography Sensor ^64^Cu-Macrin in Mice, Rabbits, and Pigs. Circ. Cardiovasc. Imaging.

[68] Gustafson H.H., Holt-Casper D., Grainger D.W., Ghandehari H. (2015). Nanoparticle Uptake: The Phagocyte Problem. Nano Today.

[69] Weissleder R., Nahrendorf M., Pittet M.J. (2014). Imaging macrophages with nanoparticles. Nat. Mater..

[70] Kooi M.E., Cappendijk V.C., Cleutjens K.B.J.M., Kessels A.G.H., Kitslaar P.J.E.H.M., Borgers M., Frederik P.M., Daemen M., Van Engelshoven J.M.A. (2003). Accumulation of ultrasmall superparamagnetic particles of iron oxide in human atherosclerotic plaques can be detected by in vivo magnetic resonance imaging. Circulation.

[71] Majmudar M.D., Yoo J., Keliher E.J., Truelove J.J., Iwamoto Y., Sena B., Dutta P., Borodovsky A., Fitzgerald K., Di Carli M.F. (2013). Polymeric nanoparticle PET/MR imaging allows macrophage detection in atherosclerotic plaques. Circ. Res..

[72] Liu Y., Abendschein D., Woodard G.E., Rossin R., McCommis K., Zheng J., Welch M.J., Woodard P.K. (2010). Molecular Imaging of Atherosclerotic Plaque with ^64^Cu-Labeled Natriuretic Peptide and PET. J. Nucl. Med..

[73] Detering L., Abdilla A., Luehmann H.P., Williams J.W., Huang L.-H., Sultan D., Elvington A., Heo G.S., Woodard P.K., Gropler R.J. (2021). CC Chemokine Receptor 5 Targeted Nanoparticles Imaging the Progression and Regression of Atherosclerosis Using Positron Emission Tomography/Computed Tomography. Mol. Pharm..

[74] Jamar F., Buscombe J., Chiti A., Christian P.E., Delbeke D., Donohoe K.J., Israel O., Martin-Comin J., Signore A. (2013). EANM/SNMMI guideline for ^18^F-FDG use in inflammation and infection. J. Nucl. Med..

[75] Tsan M.-F. (1985). Mechanism of Gallium-67 Accumulation in Inflammatory Lesions. J. Nucl. Med. Soc. Nucl. Med..

[76] Gemmel F., Van den Wyngaert H., Love C., Welling M.M., Gemmel P., Palestro C.J. (2012). Prosthetic joint infections: Radionuclide state-of-the-art imaging. Eur. J. Nucl. Med. Mol. Imaging.

[77] Lindsay M.J., Siegel B.A., Tunis S.R., Hillner B.E., Shields A.F., Carey B.P., Coleman R.E. (2007). The National Oncologic PET Registry: Expanded medicare coverage for PET under coverage with evidence development. AJR Am. J. Roentgenol..

[78] Celermajer D.S. (1998). Noninvasive detection of atherosclerosis. N. Engl. J. Med..

[79] Kubota R., Yamada S., Kubota K., Ishiwata K., Tamahashi N., Ido T. (1992). Intratumoral Distribution of Fluorine-18-Fluorodeoxyglucose In Vivo: High Accumulation in Macrophages and Granulation Tissues Studied by Microautoradiography. J. Nucl. Med. Soc. Nucl. Med..

[80] Gamelli R.L., Liu H., He L.K., Hofmann C.A. (1996). Augmentations of glucose uptake and glucose transporter-1 in macrophages following thermal injury and sepsis in mice. J. Leukoc. Biol..

[81] Mochizuki T., Tsukamoto E., Kuge Y., Kanegae K., Zhao S., Hikosaka K., Hosokawa M., Kohanawa M., Tamaki N. (2001). FDG Uptake and Glucose Transporter Subtype Expressions in Experimental Tumor and Inflammation Models. J. Nucl. Med. Soc. Nucl. Med..

[82] Tawakol A., Migrino R.Q., Hoffmann U., Abbara S., Houser S., Gewirtz H., Muller J.E., Brady T.J., Fischmanb A.J. (2005). Noninvasive in vivo measurement of vascular inflammation with F-18 fluorodeoxyglucose positron emission tomography. J. Nucl. Cardiol..

[83] Van der Wal A.C., Becker A.E., van der Loos C.M., Das P.K. (1994). Site of intimal rupture or erosion of thrombosed coronary atherosclerotic plaques is characterized by an inflammatory process irrespective of the dominant plaque morphology. Circulation.

[84] Libby P. (2002). Inflammation in atherosclerosis. Nature.

[85] Zhang Z., Machac J., Helft G., Worthley S.G., Tang C.Y., Zaman A.G., Rodriguez O.J., Buchsbaum M.S., Fuster V., Badimon J.J. (2006). Non-invasive imaging of atherosclerotic plaque macrophage in a rabbit model with F-18 FDG PET: A histopathological correlation. BMC Nucl. Med..

[86] Liu J., Kerwin W.S., Caldwell J.H., Ferguson M.S., Hippe D.S., Alessio A.M., Martinez-Malo V., Pimentel K., Miyaoka R.S., Kohler T.R. (2016). High resolution FDG-microPET of carotid atherosclerosis: Plaque components underlying enhanced FDG uptake. Int. J. Cardiovasc. Imaging.

[87] Tawakol A., Migrino R.Q., Bashian G.G., Bedri S., Vermylen D., Cury R.C., Yates D., LaMuraglia G.M., Furie K., Houser S. (2006). In vivo ^18^F-fluorodeoxyglucose positron emission tomography imaging provides a noninvasive measure of carotid plaque inflammation in patients. J. Am. Coll. Cardiol..

[88] Cocker M.S., Spence J.D., Hammond R., Dekemp R.A., Lum C., Wells G., Bernick J., Hill A., Nagpal S., Stotts G. (2018). [^18^F]-Fluorodeoxyglucose PET/CT imaging as a marker of carotid plaque inflammation, Comparison to immunohistology and relationship to acuity of events. Int. J. Cardiol..

[89] Figueroa Amparo L., Subramanian Sharath S., Cury Ricardo C., Truong Quynh A., Gardecki Joseph A., Tearney Guillermo J., Hoffmann U., Brady T.J., Tawakol A. (2012). Distribution of Inflammation within Carotid Atherosclerotic Plaques with High-Risk Morphological Features. Circ. Cardiovasc. Imaging.

[90] Hyafil F., Schindler A., Sepp D., Obenhuber T., Bayer-Karpinska A., Boeckh-Behrens T., Höhn S., Hacker M., Nekolla S.G., Rominger A. (2016). High-risk plaque features can be detected in non-stenotic carotid plaques of patients with ischaemic stroke classified as cryptogenic using combined (^18^)F-FDG PET/MR imaging. Eur. J. Nucl. Med. Mol. Imaging.

[91] Rominger A., Saam T., Wolpers S., Cyran C.C., Schmidt M., Foerster S., Nikolaou K., Reiser M.F., Bartenstein P., Hacker M. (2009). ^18^F-FDG PET/CT Identifies Patients at Risk for Future Vascular Events in an Otherwise Asymptomatic Cohort with Neoplastic Disease. J. Nucl. Med..

[92] Kelly P.J., Camps-Renom P., Giannotti N., Martí-Fàbregas J., Murphy S., McNulty J., Barry M., Barry P., Calvet D., Coutts S.B. (2019). Carotid Plaque Inflammation Imaged by ^18^F-Fluorodeoxyglucose Positron Emission Tomography and Risk of Early Recurrent Stroke. Stroke.

[93] Mb M.M., Merwick A., Mb O.C.S., Hannon N., Foran P., Grant T., Dolan E., Moroney J., Murphy S., O’Rourke K. (2012). Carotid plaque inflammation on ^18^F-fluorodeoxyglucose positron emission tomography predicts early stroke recurrence. Ann. Neurol..

[94] Wu Y.-W., Kao H.-L., Huang C.-L., Chen M.-F., Lin L.-Y., Wang Y.-C., Lin Y.-H., Lin H.-J., Tzen K.-Y., Yen R.-F. (2012). The effects of 3-month atorvastatin therapy on arterial inflammation, calcification, abdominal adipose tissue and circulating biomarkers. Eur. J. Nucl. Med. Mol. Imaging.

[95] Ishii H., Nishio M., Takahashi H., Aoyama T., Tanaka M., Toriyama T., Tamaki T., Yoshikawa D., Hayashi M., Amano T. (2010). Comparison of atorvastatin 5 and 20 mg/d for reducing F-18 fluorodeoxyglucose uptake in atherosclerotic plaques on positron emission tomography/computed tomography: A randomized, investigator-blinded, open-label, 6-month study in Japanese adults scheduled for percutaneous coronary intervention. Clin. Ther..

[96] Hyafil F., Feldman L., Le Guludec D., Fayad Z.A. (2012). Evaluating efficacy of pharmaceutical interventions in atherosclerosis: Role of magnetic resonance imaging and positron emission tomography. Mt. Sinai J. Med..

[97] Tahara N., Kai H., Ishibashi M., Nakaura H., Kaida H., Baba K., Hayabuchi N., Imaizumi T. (2006). Simvastatin attenuates plaque inflammation: Evaluation by fluorodeoxyglucose positron emission tomography. J. Am. Coll. Cardiol..

[98] Rudd J.H., Myers K.S., Bansilal S., Machac J., Rafique A., Farkouh M., Fuster V., Fayad Z.A. (2007). (^18^)Fluorodeoxyglucose positron emission tomography imaging of atherosclerotic plaque inflammation is highly reproducible: Implications for atherosclerosis therapy trials. J. Am. Coll. Cardiol..

[99] Tawakol A., Fayad Z.A., Mogg R., Alon A., Klimas M.T., Dansky H., Subramanian S.S., Abdelbaky A., Rudd J.H.F., Farkouh M.E. (2013). Intensification of statin therapy results in a rapid reduction in atherosclerotic inflammation: Results of a multicenter fluorodeoxyglucose-positron emission tomography/computed tomography feasibility study. J. Am. Coll. Cardiol..

[100] Tomas L., Edsfeldt A., Mollet I., Matic L.P., Prehn C., Adamski J., Paulsson-Berne G., Hedin U., Nilsson J., Bengtsson E. (2018). Altered metabolism distinguishes high-risk from stable carotid atherosclerotic plaques. Eur. Heart J..

[101] Ridker P.M., Everett B.M., Thuren T., MacFadyen J.G., Chang W.H., Ballantyne C., Fonseca F., Nicolau J., Koenig W., Anker S.D. (2017). Antiinflammatory Therapy with Canakinumab for Atherosclerotic Disease. N. Engl. J. Med..

[102] Gao X., Qian P., Cen D., Hong W., Peng Q., Xue M. (2018). Synthesis of phosphatidylcholine in rats with oleic acid-induced pulmonary edema and effect of exogenous pulmonary surfactant on its De Novo synthesis. PLoS ONE.

[103] Insull W., Bartsch G.E. (1966). Cholesterol, triglyceride, and phospholipid content of intima, media, and atherosclerotic fatty streak in human thoracic aorta. J. Clin. Investig..

[104] Boggs K.P., Rock C.O., Jackowski S. (1995). Lysophosphatidylcholine and 1-*O*-Octadecyl-2-*O*-Methyl-rac-Glycero-3-Phosphocholine Inhibit the CDP-Choline Pathway of Phosphatidylcholine Synthesis at the CTP, Phosphocholine Cytidylyltransferase Step (∗). J. Biol. Chem..

[105] Haeffner E.W. (1975). Studies on choline permeation through the plasma membrane and its incorporation into phosphatidyl choline of Ehrlich-Lettré-ascites tumor cells in vitro. Eur. J. Biochem..

[106] Schmid D.T., John H., Zweifel R., Cservenyak T., Westera G., Goerres G.W., Von Schulthess G.K., Hany T. (2005). FFluorocholine PET/CT in patients with prostate cancer: Initial experience. Radiology.

[107] Yoshimoto M., Waki A., Obata A., Furukawa T., Yonekura Y., Fujibayashi Y. (2004). Radiolabeled choline as a proliferation marker: Comparison with radiolabeled acetate. Nucl. Med. Biol..

[108] Wyss M.T., Weber B., Honer M., Späth N., Ametamey S.M., Westera G., Bode B., Kaim A.H., Buck A. (2004). ^18^F-choline in experimental soft tissue infection assessed with autoradiography and high-resolution PET. Eur. J. Nucl. Med. Mol. Imaging.

[109] DeGrado T.R., Coleman R.E., Wang S., Baldwin S.W., Orr M.D., Robertson C.N., Polascik T.J., Price D.T. (2001). Synthesis and evaluation of ^18^F-labeled choline as an oncologic tracer for positron emission tomography: Initial findings in prostate cancer. Cancer Res..

[110] Velikyan I. (2018). Prospective of ^68^Ga Radionuclide Contribution to the Development of Imaging Agents for Infection and Inflammation. Contrast Media Mol. Imaging.

[111] Bozkurt M.F., Virgolini I., Balogova S., Beheshti M., Rubello D., Decristoforo C., Ambrosini V., Kjaer A., Delgado-Bolton R., Kunikowska J. (2017). Guideline for PET/CT imaging of neuroendocrine neoplasms with ^68^Ga-DOTA-conjugated somatostatin receptor targeting peptides and ^18^F-DOPA. Eur. J. Nucl. Med. Mol. Imaging.

[112] Brazeau P., Vale W., Burgus R., Ling N., Butcher M., Rivier J., Guillemin R. (1973). Hypothalamic polypeptide that inhibits the secretion of immunoreactive pituitary growth hormone. Science.

[113] Moore K.J., Tabas I. (2011). Macrophages in the pathogenesis of atherosclerosis. Cell.

[114] Rinne P., Hellberg S., Kiugel M., Virta J., Li X.-G., Käkelä M., Helariutta K., Luoto P., Liljenbäck H., Hakovirta H. (2016). Comparison of Somatostatin Receptor 2-Targeting PET Tracers in the Detection of Mouse Atherosclerotic Plaques. Mol. Imaging Biol..

[115] Li X., Bauer W., Kreissl M.C., Weirather J., Bauer E., Israel I., Richter D., Riehl G., Buck A., Samnick S. (2013). Specific somatostatin receptor II expression in arterial plaque: (^68^)Ga-DOTATATE autoradiographic, immunohistochemical and flow cytometric studies in apoE-deficient mice. Atherosclerosis.

[116] Armani C., Catalani E., Balbarini A., Bagnoli P., Cervia D. (2007). Expression, pharmacology, and functional role of somatostatin receptor subtypes 1 and 2 in human macrophages. J. Leukoc. Biol..

[117] Dalm V.A.S.H., Van Hagen P.M., Van Koetsveld P.M., Achilefu S., Houtsmuller A.B., Pols D., Van Der Lely A.-J., Lamberts S.W.J., Hofland L.J. (2003). Expression of somatostatin, cortistatin, and somatostatin receptors in human monocytes, macrophages, and dendritic cells. Am. J. Physiol.-Endocrinol. Metab. Am. Physiol. Soc..

[118] Li X., Samnick S., Lapa C., Israel I., Buck A.K., Kreissl M.C., Bauer W. (2012). ^68^Ga-DOTATATE PET/CT for the detection of inflammation of large arteries: Correlation with18F-FDG, calcium burden and risk factors. EJNMMI Res..

[119] Malmberg C., Ripa R.S., Johnbeck C.B., Knigge U., Langer S.W., Mortensen J., Oturai P., Loft A., Hag A.M., Kjaer A. (2015). ^64^Cu-DOTATATE for Noninvasive Assessment of Atherosclerosis in Large Arteries and Its Correlation with Risk Factors: Head-to-Head Comparison with ^68^Ga-DOTATOC in 60 Patients. J. Nucl. Med. Soc. Nucl. Med..

[120] Mojtahedi A., Alavi A., Thamake S., Amerinia R., Ranganathan D., Tworowska I., Delpassand E.S. (2015). Assessment of vulnerable atherosclerotic and fibrotic plaques in coronary arteries using (^68^)Ga-DOTATATE PET/CT. Am. J. Nucl. Med. Mol. Imaging.

[121] Schatka I., Wollenweber T., Haense C., Brunz F., Gratz K.F., Bengel F.M. (2013). Peptide receptor-targeted radionuclide therapy alters inflammation in atherosclerotic plaques. J. Am. Coll. Cardiol..

[122] Pedersen S.F., Sandholt B.V., Keller S.H., Hansen A.E., Clemmensen A.E., Sillesen H., Højgaard L., Ripa R.S., Kjær A. (2015). ^64^Cu-DOTATATE PET/MRI for Detection of Activated Macrophages in Carotid Atherosclerotic Plaques: Studies in Patients Undergoing Endarterectomy. Arter. Thromb. Vasc. Biol..

[123] Virmani R., Kolodgie F.D., Burke A.P., Farb A., Schwartz S.M. (2000). Lessons from sudden coronary death: A comprehensive morphological classification scheme for atherosclerotic lesions. Arter. Thromb. Vasc. Biol..

[124] Nakahara T., Dweck M.R., Narula N., Pisapia D., Narula J., Strauss H.W. (2017). Coronary Artery Calcification: From Mechanism to Molecular Imaging. JACC Cardiovasc. Imaging.

[125] Lee R., Kim J., Paeng J.C., Byun J.W., Cheon G.J., Lee D.S., Chung J.-K., Kang K.W. (2018). Measurement of ^68^Ga-DOTATOC Uptake in the Thoracic Aorta and Its Correlation with Cardiovascular Risk. Nucl. Med. Mol. Imaging.

[126] Velikyan I. (2013). Prospective of ^68^Ga-radiopharmaceutical development. Theranostics.

[127] Arend W.P., Michel B.A., Bloch D.A., Hunder G.G., Do L.H.C., Edworthy S.M., Fauci A.S., Leavitt R.Y., Lie J.T., Lightfoot R.W. (1990). The American College of Rheumatology 1990 criteria for the classification of Takayasu arteritis. Arthritis Rheum..

[128] Hunder G.G., Bloch D.A., Michel B.A., Stevens M.B., Arend W.P., Calabrese L.H., Edworthy S.M., Fauci A.S., Leavitt R.Y., Lie J.T. (1990). The American College of Rheumatology 1990 criteria for the classification of giant cell arteritis. Arthritis Rheum..

[129] Slart R.H.J.A., Writing group, Reviewer group, Members of EANM Cardiovascular, Members of EANM Infection & Inflammation, Members of Committees, SNMMI Cardiovascular, SNMMI Cardiovascular, Members of Council, PET Interest Group, Members of ASNC (2018). FDG-PET/CT(A) imaging in large vessel vasculitis and polymyalgia rheumatica: Joint procedural recommendation of the EANM, SNMMI, and the PET Interest Group (PIG), and endorsed by the ASNC. Eur. J. Nucl. Med. Mol. Imaging.

[130] Kerr G.S., Hallahan C.W., Giordano J., Leavitt R.Y., Fauci A.S., Rottem M., Hoffman G.S. (1994). Takayasu arteritis. Ann. Intern. Med..

[131] Cheng Y., Lv N., Wang Z., Chen B., Dang A. (2013). 18-FDG-PET in assessing disease activity in Takayasu arteritis: A meta-analysis. Clin. Exp. Rheumatol..

[132] Besson F.L., Parienti J.-J., Bienvenu B., Prior J.O., Costo S., Bouvard G., Agostini D. (2011). Diagnostic performance of ^18^F-fluorodeoxyglucose positron emission tomography in giant cell arteritis: A systematic review and meta-analysis. Eur. J. Nucl. Med. Mol. Imaging.

[133] Soussan M., Nicolas P., Schramm C., Katsahian S., Pop G., Fain O., Mekinian A. (2015). Management of large-vessel vasculitis with FDG-PET: A systematic literature review and meta-analysis. Medicine.

[134] Tatò F., Hoffmann U. (2008). Giant cell arteritis: A systemic vascular disease. Vasc. Med..

[135] Alessi M.C., Juhan-Vague I., Declerck P.J., Collen D. (1991). Molecular forms of plasminogen activator inhibitor-1 (PAI-1) and tissue-type plasminogen activator (t-PA) in human plasma. Thromb. Res..

[136] Löffler C., Hoffend J., Benck U., Krämer B.K., Bergner R. (2017). The value of ultrasound in diagnosing extracranial large-vessel vasculitis compared to FDG-PET/CT: A retrospective study. Clin. Rheumatol..

[137] Einspieler I., Thürmel K., Pyka T., Eiber M., Wolfram S., Moog P., Reeps C., Essler M. (2015). Imaging large vessel vasculitis with fully integrated PET/MRI: A pilot study. Eur. J. Nucl. Med. Mol. Imaging.

[138] Chatterjee S., Flamm S.D., Tan C.D., Rodriguez E.R. (2014). Clinical diagnosis and management of large vessel vasculitis: Giant cell arteritis. Curr. Cardiol. Rep..

[139] Schmall J.P., Karp J.S., Alavi A. (2019). The Potential Role of Total Body PET Imaging in Assessment of Atherosclerosis. PET Clin..

[140] De Boysson H., Dumont A., Liozon E., Lambert M., Boutemy J., Maigné G., Silva N.M., Sultan A., Ly K.H., Aide N. (2017). Giant-cell arteritis: Concordance study between aortic CT angiography and FDG-PET/CT in detection of large-vessel involvement. Eur. J. Nucl. Med. Mol. Imaging.

[141] Misra D.P., Shenoy S.N. (2017). Cardiac involvement in primary systemic vasculitis and potential drug therapies to reduce cardiovascular risk. Rheumatol. Int..

[142] Scholtens A.M., Verberne H.J., Budde R.P.J., Lam M.G.E.H. (2016). Additional Heparin Preadministration Improves Cardiac Glucose Metabolism Suppression over Low-Carbohydrate Diet Alone in ^18^F-FDG PET Imaging. J. Nucl. Med. Soc. Nucl. Med..

[143] Dorbala S., Di Carli M.F., Delbeke D., Abbara S., DePuey E.G., Dilsizian V., Forrester J., Janowitz W., Kaufmann P.A., Mahmarian J. (2013). SNMMI/ASNC/SCCT Guideline for Cardiac SPECT/CT and PET/CT 1.0. J. Nucl. Med. Soc. Nucl. Med..

[144] Ben-Haim S., Kupzov E., Tamir A., Israel O. (2004). Evaluation of *18*F-FDG Uptake and Arterial Wall Calcifications Using *18*F-FDG PET/CT. J. Nucl. Med. Soc. Nucl. Med..

[145] Nielsen B.D., Gormsen L.C., Hansen I.T., Keller K.K., Therkildsen P., Hauge E.-M. (2018). Three days of high-dose glucocorticoid treatment attenuates large-vessel ^18^F-FDG uptake in large-vessel giant cell arteritis but with a limited impact on diagnostic accuracy. Eur. J. Nucl. Med. Mol. Imaging.

[146] Stellingwerff M.D., Brouwer E., Lensen K.-J.D.F., Rutgers A., Arends S., van der Geest K.S.M., Glaudemans A.W.J.M., Slart R.H.J.A. (2015). Different Scoring Methods of FDG PET/CT in Giant Cell Arteritis: Need for Standardization. Medicine.

